# Estrategia para la gobernanza de datos abiertos de salud: un cambio de paradigma en los sistemas de información

**DOI:** 10.26633/RPSP.2017.27

**Published:** 2017-03-23

**Authors:** Marcelo D’Agostino, Myrna Marti, Felipe Mejía, Gerardo de Cosio, Gladys Faba

**Affiliations:** 1 Unidad de Análisis e Información en Salud, Departamento de Enfermedades Transmisibles y Análisis de Salud Organización Panamericana de la Salud/Organización Mundial de la Salud Washington D.C Estados Unidos de América Unidad de Análisis e Información en Salud, Departamento de Enfermedades Transmisibles y Análisis de Salud, Organización Panamericana de la Salud/Organización Mundial de la Salud, Washington D.C., Estados Unidos de América.; 2 Oficina de Gestión del Conocimiento, Bioética e Investigación Organización Panamericana de la Salud/Organización Mundial de la Salud Buenos Aires Argentina Oficina de Gestión del Conocimiento, Bioética e Investigación, Organización Panamericana de la Salud/Organización Mundial de la Salud, Buenos Aires, Argentina.; 3 Consultor internacional Consultor internacional Bogotá Colombia Consultor internacional, Bogotá, Colombia.; 4 Consultora internacional Tlayacapán México Consultora internacional Tlayacapán, México.

**Keywords:** Base de datos, acceso abierto, análisis de datos, confidencialidad, sistemas de información, fuentes de datos, interpretación estadística de datos, minería de datos, Database, open access, data analysis, confidentiality, information systems, data sources, data mining, Base de dados, acesso aberto, análise de dados, confidencialidade, sistemas de informação, fontes de dados, mineração de dados

## Abstract

Para la formulación o revisión de políticas, planificación, innovación, investigación, medición del progreso, barreras u oportunidades, se requiere de acceso abierto a datos confiables, información y conocimiento calificado. Esto forma parte de los principios del Código Sanitario Panamericano y de la Constitución de la Organización Panamericana de la Salud. Entre 1950 y 1978, el tema de recolección de datos de salud, en particular sobre estadísticas vitales, se manifestó de manera expresa en cada una de las primeras siete ediciones de la publicación conocida como “Salud en las Américas”. Sin embargo, y a pesar de reconocerse una mejora considerable en la calidad de los datos, se reconoce desde el inicio de los 80 que los sistemas de información en salud de los países requieren de una modernización constante para proporcionar datos de salud con mejor calidad, cobertura y oportunidad para la toma de decisiones informadas. A partir del auge de Internet, el creciente acceso a tecnologías de información y comunicación, y hoy en día con las redes sociales, el aumento de contenidos disponibles se ha masificado y crece de manera exponencial, no estructurada, no clasificada y, muchas veces, sin control. Esta situación obliga a las instituciones de salud a adoptar estrategias y estándares para la gestión de datos abiertos que permitan fortalecer calidad, confidencialidad y seguridad de los mismos. Se propone una estrategia de gobernanza de datos de salud con herramientas, conceptos y recomendaciones que permitirán a los países generar datos abiertos y de mayor calidad, así como confiables y seguros.

## Abstract (en)

Policy development and review, planning, innovation, research, and the measurement of progress, barriers, and opportunities require open access to reliable data, information, and expert knowledge. This is part of the principles of the Pan American Sanitary Code and the Constitution of the Pan American Health Organization. Between 1950 and 1978, the subject of health data collection, in particular vital statistics, was expressly addressed in each of the first seven editions of Health in the Americas. However, despite considerable improvement in data quality, since the early 1980s it has been recognized that countries’ health information systems require constant modernization in order to provide health data with better quality, coverage, and timeliness for informed decision-making. With the rise of the Internet, growing access to information and communications technologies––and today, with social networks, the increase in available content––has become massive and is growing in an exponential, unstructured, unclassified, and often uncontrolled manner. This situation forces health institutions to adopt strategies and standards for the management of open data that make it possible to strengthen their quality, confidentiality, and security. We propose a strategy for the governance of health data using tools, concepts, and recommendations that will enable countries to generate open, higher-quality, reliable, and secure data.

## RESUMO

A formulação ou revisão de políticas, o planejamento, a inovação, a pesquisa e a medição do progresso, barreiras ou oportunidades requerem o acesso aberto a dados confiáveis, informações e conhecimentos qualificados. Isto faz parte dos princípios do Código Sanitário PanAmericano e da Constituição da Organização PanAmericana da Saúde. Entre 1950 e 1978, a questão da compilação de dados de saúde, em particular sobre estatísticas vitais, manifestou-se expressamente em cada uma das sete primeiras edições da publicação conhecida como “Saúde nas Américas”. No entanto, embora tenha sido observada uma melhoria considerável na qualidade dos dados, existe o reconhecimento, desde o início da década de 1980, de que os sistemas de informação em saúde dos países precisam ser modernizados constantemente para proporcionar dados de saúde com melhor qualidade, cobertura e oportunidades para a tomada de decisões bem embasadas. Desde o despontar da Internet, com o crescente acesso a tecnologias da informação e comunicação, e hoje em dia com as redes sociais, o volume de conteúdos disponíveis tem se massificado e cresce exponencialmente de forma não estruturada, não classificada e, muitas vezes, descontrolada. Esta situação obriga as instituições de saúde a adotar estratégias e normas para a gestão de dados abertos que permitam fortalecer a qualidade, a confidencialidade e a segurança dos dados. Propomos aqui uma estratégia de governança dos dados de saúde por meio de ferramentas, conceitos e recomendações que permitirão aos países gerar dados abertos e de maior qualidade, além de confiáveis e seguros.

En 1954 tuvo lugar en Santiago de Chile la Conferencia Sanitaria Panamericana (CSP), que es la autoridad suprema de la Organización Panamericana de la Salud (OPS) y se reúne cada cinco años para determinar las políticas generales de esta. En aquella ocasión, se presentó el primer informe regional del estado de situación de la salud de las Américas bajo el título *Summary of reports on health conditions in the Americas 1950–1953*, una revisión del documento CSP14/17 presentado el 10 de setiembre de 1954. Se solicitó a cada país miembro que completara un informe cuadrienal sobre las condiciones de salud, preferiblemente de naturaleza estadística. Quedó establecido que el progreso de la salud dependería, en gran medida, de la medición de los problemas a partir del acceso a datos de mayor calidad que permitan una planificación estratégica y efectiva en salud pública. Dicho proceso se ha desarrollado, de manera ininterrumpida, desde 1950 hasta la fecha, con 15 ediciones de la que es la principal publicación de la Organización Panamericana de la Salud/Organización Mundial de la Salud (OPS/OMS), conocida como “Salud en Las Américas” (cuadro 1).

Los datos publicados en el primer informe (1950–53) incluyeron información de censos, de estadísticas vitales y de sistemas de notificación de casos, así como una breve descripción de los programas de control de enfermedades transmisibles, la cantidad de personal empleado en los servicios de salud, la organización de los servicios nacionales de salud y varios aspectos de los programas de saneamiento (1). Con el pasar de los años y la evolución de las tecnologías de información, la OPS ha ido modificando el contenido y el formato de la publicación, con el fin de adaptarla a la situación actual de la región que presenta, entre otras, una mayor complejidad en los sistemas y servicios de salud, así como un crecimiento acelerado en la generación de datos, estructurados o no, y también de información, basada en evidencia o no.

**CUADRO 1.** Cronología de publicación de datos de los países miembros de la Organización Panamericana d ela Salud, 1950–2012

Número	Período	Año de publicación	Conferencia Sanitaria Panamericana	Título	Director(a) de la OPS
1	1950–1953	1956	14	Resumen de los Informes sobre las Condiciones de Salud en las Américas	Fred Soper
2	1954–1956	1958	15	Resumen de los Informes cuadrienales sobre las Condiciones de Salud en las Américas	Fred Soper
3	1957–1960	1962	16	Resumen de los Informes cuadrienales sobre las Condiciones de Salud en las Américas	Abraham Horwitz
4	1961–1964	1966	17	Condiciones de salud en las Américas	Abraham Horwitz
5	1965–1968	1970	18	Condiciones de salud en las Américas	Abraham Horwitz
6	1969–1972	1974	19	Condiciones de salud en las Américas	Abraham Horwitz
7	1973–1976	1978	20	Condiciones de salud en las Américas	Héctor Acuña
8	1977–1980	1982	21	Condiciones de salud en las Américas	Héctor Acuña
9	1981–1984	1986	22	Condiciones de salud en las Américas Volumen I/II	Carlyle G. de Macedo
10	1985–1988	1990	23	Condiciones de salud en las Américas Volumen I/II	Carlyle G. de Macedo
11	1989–1992	1994	24	Condiciones de salud en las Américas Volumen I/II	Carlyle G. de Macedo
12	1993–1996	1998	25	Salud en las Américas Volumen I/II	George Alleyne
13	1997–2000	2002	26	Salud en las Américas Volumen I/II	George Alleyne
14	2001–2005	2007	27	Salud en las Américas Volumen I/II	Mirta Roses
15	2006–2010	2012	28	Salud en las Américas 2012Visión regional y perfiles de país	Mirta Roses

Desde hace más de medio siglo, los gobiernos de la región de las Américas destacaron, durante las reuniones del Consejo Directivo de la OPS, la importancia del uso datos de salud para la planeación de acciones a nivel de país, subregional, regional y global. Un ejemplo de ello se ve cuando, en el período 1969–1972, hubo la creencia de que los datos de salud de los países serían de gran utilidad para la realización de un Plan Decenal de Salud, y que podría ser, además, parte integral de la Carta de Punta del Este (2). Asimismo, se planteó la importancia y necesidad de ampliar los programas de la OPS en este campo fundamental, y en la firme intención de los gobiernos de utilizar y mejorar sus datos en cantidad y calidad de acuerdo a la situación de cada país (2).

Entre 1950 y 1978, los países de la región de las Américas dieron especial importancia al tema de estadísticas vitales, incluidas en un capítulo aparte en las primeras siete ediciones de la publicación. Sin embargo, y a pesar de reconocerse una mejora considerable en la calidad de los datos, al inicio de la década de los 80 se reconoce que los sistemas de información existentes en los países requieren de una modernización para proporcionar datos de salud con mejor calidad, cobertura y oportunidad para la toma de decisiones informadas. El aumento masivo de datos e información disponibles en la web abre un escenario más complejo, debido al gran volumen de datos y por la rapidez de este crecimiento, lo cual no permite que los usuarios de esta información estén suficientemente preparados para consultarla y hacer un mejor uso de ella. Vivimos, hoy en día, en un mundo donde es posible acceder a todo tipo de contenidos, de mayor o menor calidad, a través de múltiples caminos; sin embargo, tener las competencias para entender su verdadero valor es un privilegio que aún no está al alcance de todas las personas (3–5).

De igual modo, para cualquier trabajador de salud pública, en el maremágnum de datos e información que se manejan hoy en día por la web, dominar los métodos y las herramientas de búsquedas que sirven para identificar lo que es realmente útil se ha convertido en una de las habilidades más demandadas en el mundo laboral (6). Ante este escenario complejo e irreversible, los autores recomiendan el establecimiento de una estrategia de gobernanza de datos abiertos que otorga herramientas, conceptos y acciones que permitirán a las instituciones de salud generar datos de mayor calidad, confiables, seguros y enmarcados en un modelo renovado de sistemas de información en salud y que aseguren privacidad y confidencialidad de los datos personales de los ciudadanos. Dentro de los principales aspectos positivos de tener datos abiertos, entre muchos otros, se destacan: a) la reducción de brechas informacionales y fortalecimiento de vínculos entre las instituciones de gobierno y todas aquellas que forman parte de los sistemas de salud, incluidos los representantes de la sociedad civil y los pacientes, b) el fortalecimiento de los procesos de investigación y traducción del conocimiento en las instituciones académicas, científicas y gubernamentales, c) la formulación de políticas públicas basadas en evidencias, d) la mejor preparación o detección temprana de posibles brotes de enfermedades y e) el desarrollo de campañas de comunicación social más efectivas con mejor uso de las tecnologías de información y redes sociales (7–9). El movimiento de datos abiertos pone presión a los gobiernos para liberar grandes cantidades de información, asumiendo que cualquier tipo de datos controlados por instituciones gubernamentales deben ser datos cuya propiedad es de la población. Sin embargo, es fundamental que los gobiernos tengan normas y procedimientos basados en principios técnicos, de privacidad y éticos, que dictaminen qué datos pueden ser liberados públicamente (10).

Concebimos la gobernanza de datos como la apropiación de los datos por parte de quienes toman decisiones en los diversos niveles de las organizaciones gracias, por una parte, a la precisión y transparencia que estas alcanzan para apoyar decisiones efectivas y, por otra parte, a los procedimientos de control establecidos como parte de las políticas que aseguran que la información sea precisa, coherente y accesible. El proceso de gobernar datos abiertos exige aumentar la calidad de su producción original, mejorar y ampliar su mapeo y categorización, así como asegurar el cumplimiento de estrictos estándares éticos que aseguren su privacidad, confidencialidad y uso responsable (10).

## DESCRIPCIÓN GENERAL Y ALCANCE DE LA ESTRATEGIA

Esta estrategia es un instrumento clave para el desarrollo e implementación organizacional de políticas, estándares, normas y procedimientos, y herramientas de gestión de datos abiertos que actúen como mecanismos que aseguran mayor calidad en la producción y recolección de los datos, así como para el establecimiento de estándares internacionales para normalización e interoperabilidad. También sirve como sistema transparente de toma de decisiones y rendición de cuentas en los procesos de gestión de datos de salud (11) y, a su vez, como un mecanismo que promueve el concepto de la importancia de tener una ciudadanía informada y participativa de los temas de salud. Lo anterior se refiere a la gestión de la disponibilidad, facilidad de uso, integridad, privacidad y seguridad de los datos de salud, tanto interna como externa, gestionados por la institución y que se formaliza a través de un conjunto de regulaciones, políticas, principios rectores, normas y procedimientos que abarcan el ciclo de vida completo de los datos: adquisición, utilización, difusión y eliminación. El ámbito de aplicación de esta estrategia abarca todos aquellos datos y otros contenidos relacionados con los que interactúan y que están sujetos a la autoridad administrativa de la institución. Las instituciones pueden ser productoras, usuarias o intermediarias de datos de salud. Es recomendable que todas aquellas que gestionen datos y otros contenidos relacionados se adhieran en forma voluntaria a las normas y requisitos establecidos por las instituciones rectoras de salud de una nación. Varios países del mundo han formulado políticas y planes de apertura de datos, considerando la información como un recurso nacional valioso y un activo estratégico. Un ejemplo de ello en la región de las Américas es la política de datos abiertos de Estados Unidos de América, así como desarrollos subregionales en el Caribe, como el *Caribbean Open Institute*y la Iniciativa Latinoamericana por los Datos Abiertos (ILDA). La Unión Europea tiene un grado de avance importante en este tema y se encuentra concentrado en el *European Union Open Data Portal*.

El objetivo final de la estrategia no es solo proteger datos, sino también garantizar mayor calidad en su producción y recolección y permitir una gestión eficaz y eficiente de ellos bajo estrictos principios de privacidad, seguridad y confidencialidad, para tomar mejores decisiones y formular mejores políticas que beneficien a la salud de la población, con una reducción de la probabilidad de un mal uso de esos datos y el daño que ese uso inadecuado puede causar. Para implementar dicha estrategia, se hace fundamental entender los principios para que un dato se considere abierto (12) (cuadro 2).

Uno de los principales objetivos de aplicar estos principios es poder evaluar hasta qué punto los datos públicos son realmente abiertos y están disponibles para quien pueda necesitarlos en el momento y formato necesarios. Además de la aplicación de estos principios, que son de carácter técnico, debe asegurarse la aplicación de todas las consideraciones éticas que promueven la apertura de todas las bases de datos.

## PRINCIPIOS RECTORES PARA UNA ESTRATEGIA EFECTIVA

Los autores consideran que la efectividad de la gobernanza de datos abiertos en el campo de la salud se fundamenta en la existencia de principios institucionales que aseguren su implementación y sostenibilidad. Estos van a regir todos los procesos asociados a la estrategia. En el cuadro 3 se presenta la descripción de estos principios.

**CUADRO 2.** Principios para datos abiertos

Completos	Primarios	Periódicos	Accesibles	Procesables	No discriminatorios	No propietarios	Sin licencia
Todos los datos públicos están disponibles y no están sujetos a restricciones de privacidad, seguridad o privilegio	Obtenidos en la fuente primaria, con el mayor nivel posible de granularidad, sin ser modificados ni agrupados	Disponibles tan pronto como sea necesario para preservar su valor	Quedan disponibles para la mayor cantidad posible de usuarios y propósitos	Están razonablemente estructurados, de forma de permitir su procesamiento automático	Están disponibles para todos, sin requisitos de registro	Están disponibles en un formato sobre el que ninguna entidad tiene control exclusivo	No están sujetos a ningún tipo de regulación de derechos, patentes o registros de marca. Se podrán permitir restricciones razonables de privacidad, seguridad o privilegio

**CUADRO 3.** Principios rectores para una estrategia efectiva

Apertura	Transparencia	Detectabilidad	Utilización en la toma de decisiones	Normalización	Seguridad	Privacidad	Calidad
Los datos de salud serán gestionados como elementos compartidos y abiertos para maximizar los valores fundamentales de la institución y para reducir potenciales riesgos	Las políticas y decisiones institucionales sobre los datos de salud serán comunicadas con claridad y transparencia a todos los potenciales productores, usuarios o intermediarios de ellos	La institución establecerá condiciones para asegurar que sus datos de salud sean abiertos y fáciles de descubrir y utilizar (13)	El objetivo final de la estrategia es garantizar la calidad y la gestión eficaz y eficiente de los datos de salud para una mejor toma de decisiones y formulación de políticas de salud pública	La gobernanza de datos de salud introducirá y apoyará los procesos de normalización e interoperabilidad de todas las bases de datos técnicos administrados por la institución	La gobernanza de datos de salud introducirá un proceso que permita el desarrollo e implementación de medidas, normas y sistemas diseñados para proteger de forma segura los datos de la salud	Aplicación de principios de la OECD: “limitación de recodiga, calidad, especificación del propósito, limitación de uso, salvaguardia de la seguridad, transparencia, participación individual, responsabilidad” (14)	Los datos deben ajustarse a las intenciones de su uso, así como su relevancia en acciones, toma de decisiones y planificación estratégica

**CUADRO 4.** Factores críticos para el éxito de una estrategia efectiva

Visión	Gobernanza	Rendición de cuentas	Confianza	Propiedad
Una estrategia de gobernanza de datos de salud fuerte y eficaz es una pieza fundacional de las instituciones de salud pública	La gobernanza de datos de salud no es un proyecto de TI, sino una responsabilidad de los titulares de los datos de salud	Un conjunto de controles y procedimientos de auditría interna debe ser implementado para garantizar el cumplimiento continuo de las normas y procedimientos de control de datos de la institución	La gobernanza de datos de salud asegura que los datos disponibles son confiables	Definición de roles y responsabilidades para todo el proceso que abarcan el ciclo de vida completo de los datos: adquisición, utilización, difusión y eliminación.

TI, tecnologías de la información.

**CUADRO 5.** Elementos más importantes de una estrategia efectiva

Programa de alfabetización digital	Conjunto de normas y procedimientos	Directorio de datos de salud	Taxonomía institucional	Glosario institucional	Esquema de permisos sobre los datos
Programa de renovación constante del saber en el uso de las tecnologías de información y comunicación aplicadas, así como en metodologías para acceso a la información y gestión del conocimiento	Conjunto de instrumentos normativos que especifican quién es responsable de la recopilación de datos, acceso, intercambio, actualización, seguimiento, auditoría, retención, privacidad y eliminación, entre otros	Catálogo de todos los conjuntos de datos técnicos de todos los niveles de la institución. Posee información de los tipos de datos, entidad responsable, el propósito de la base de datos, la ubicación digital, métodos de copias de seguridad, privilegios de TI, entre otros	Terminología normalizada para la clasficación bibliográfica de cada conjunto de datos en el directorio de datos de salud	Compendio de definiciones compartidas a ser utilizadas por el personal y usuarios de datos de salud	Conjunto de procedimientos que define cómo los conjuntos de datos de salud deben ser utilizados por el personal autorizado

TI, tecnologías de información.

## ELEMENTOS PRINCIPALES Y FACTORES CRÍTICOS PARA EL ÉXITO DE LA ESTRATEGIA

Por otro lado, existen una serie de factores y elementos que, cuando se aplican, facilitan la implementación de la estrategia, así como la consecución de los objetivos planteados en la misma. En los cuadros 4 y 5 se mencionan los factores críticos y los elementos más importantes de una estrategia de gobernanza de datos de salud.

Para lograr una implementación efectiva de la estrategia, es necesario formular a implementar una política que considere, además de lo presentado en los cuadros 4 y 5, los siguientes aspectos: 1) establecer formatos de datos que permitan maximizar el acceso, 2) proporcionar dichos formatos completos para múltiples usos, 3) eliminar cualquier restricción de acceso a la información, 4) establecer normas de licencia libre, 5) exigir la publicación de metadatos, 6) solicitar la publicación de los procesos de creación y colección de datos, 7) exigir, al máximo posible, la digitalización y distribución de materiales de archivo, 8) establecer un repositorio central dedicado a la publicación y la política de datos abiertos, 9) crear interfaces de programación de aplicaciones (API por sus siglas en inglés) públicas de acceso a la información y 10) revisar y optimizar los métodos de recolección de datos (15).

## CONSIDERACIONES FINALES

No hay duda de que nos encontramos en medio de una revolución de datos masivos sin precedentes. Además de contar con múltiples accesos a ellos, la tecnología ayuda cada vez más a procesarlos y disponemos, en cierta medida, de la posibilidad de hacer un análisis del comportamiento social en línea de las personas. Esta es una situación que solo tiende a mejorar. Estos nuevos modelos de procesamiento de datos, estructurados y no estructurados, nos abren nuevas posibilidades para el ejercicio de la Salud Pública, en especial en las áreas de prevención y predicción, ofreciendo nuevos recursos de innovación. En agosto de 2014, el Secretario General de la Organización de las Naciones Unidas, Ban Ki-moon, solicitó a un grupo de expertos independientes la elaboración de recomendaciones concretas sobre cómo la revolución de datos podría fortalecer el desarrollo sostenible. El grupo de expertos concluyó que los datos son el alma de la toma de decisiones y la materia prima para la rendición de cuentas. Se concluyó también que, sin datos de calidad que provean la información correcta en el momento necesario, se hace prácticamente imposible el diseño, monitoreo y evaluación efectiva de políticas (16). Por otro lado, un reciente editorial publicado en el *New England Journal of Medicine* sobre *Data Sharing* llama a una reflexión más profunda sobre los desafíos o limitaciones de liberar datos en el sector salud sin un enfoque adecuado (17). De la misma forma, cuando se trata de datos no estructurados, si bien estos ofrecen un gran potencial, su uso genera una serie de preocupaciones relacionadas con posible sesgo estadístico y violaciones de privacidad, entre otros aspectos. Por ello es importante formular y poner en práctica normas y procedimientos que mitiguen estos riesgos (18).

Considerando este marco sociotecnológico, estratégico y político, y luego de observar que durante más de medio siglo los gobiernos de las Américas vienen haciendo énfasis en la necesidad de mejorar la calidad de los datos de salud, se hace necesario adoptar el paradigma que nos impone la sociedad de la información, incorporando y adaptando modelos de datos abiertos que sean procesados en el marco de una estrategia institucional para la gobernanza de los mismos con calidad, privacidad y seguridad. La disponibilidad de datos abiertos de salud fortalece el concepto de ciudadanos informados y ayuda a los pacientes, prestadores de servicios de salud y a los investigadores a tomar mejores decisiones, impulsar innovaciones, detectar brechas de conocimiento e identificar intervenciones más eficientes. Los autores concluyen que la inversión en la generación y captura de datos de calidad y liberando los mismos en formato abierto permitirá a los gobiernos y las instituciones que forman parte del sistema de salud utilizar el poder de estos datos para generar políticas más costoefectivas, mejorar la calidad de la atención, disminuir costos de cuidado de la salud y facilitar la elección del paciente sobre su propia salud.

### Agradecimientos.

Los autores agradecen a los profesionales de la Unidad de Análisis e Información en Salud de la Organización Panamericana de la Salud/Organización Mundial de la Salud (OPS/OMS) por sus valiosos aportes en discusiones técnicas sobre modelos de gobernanza de datos abiertos de salud, en particular a Andrea Gerger, Patricia Soliz, Patricia Ruiz, Antonio Escamilla y Luis Gutiérrez. Los autores desean agradecer también a Tina Dannemann Purnat de la oficina de OMS para Europa.

### Declaración.

Las opiniones expresadas en este manuscrito son responsabilidad del autor y no reflejan necesariamente los criterios ni la política de la RPSP/PAJPH y/o de la OPS.
